# Dysregulated expression of microRNAs and mRNAs in pulmonary artery remodeling in ascites syndrome in broiler chickens

**DOI:** 10.18632/oncotarget.12888

**Published:** 2016-10-25

**Authors:** Ping Liu, Fei Yang, Yu Zhuang, Qingyang Xiao, Huabin Cao, Caiying Zhang, Tiancheng Wang, Huayuan Lin, Xiaoquan Guo, Guoliang Hu

**Affiliations:** ^1^ Institute of Animal Population Health, College of Animal Science and Technology, JiangXi Agricultural University, Zhimin, Nanchang Economic and Technological Development District, Nanchang, P.R. China

**Keywords:** ascites syndrome, broilers, pulmonary artery remodeling, miRNA sequencing, association analysis, Pathology Section

## Abstract

Ascites syndrome (AS), also known as pulmonary artery hypertension, remains a challenging disease that severely affects both humans and broiler chickens. Pulmonary artery remodeling presents a key step in the development of AS. In this study, we obtained pulmonary artery tissues from broilers with and without AS to perform miRNA sequencing analysis, miRNA-mRNA association analysis and pathological examinations. 29 significantly differentially expressed miRNAs were found both in known and novel miRNAs with 18 up-regulated and 11 down-regulated miRNAs. Their predicted potential targets were involved in a wide range of functional clusters as indicated via GO (Gene Ontology) and KEGG (Kyoto Encyclopedia of Genes and Genomes) analyses. The upregulation of miR-155, miR-23b-3p, miR-146b-5p and miR-146b-3p were found closely associated with the pathogenesis of pulmonary artery remodeling in AS progression. The association analysis for the miRNAs-mRNAs showed that these 29 significantly differentially expressed miRNAs regulate 162 differentially expressed target genes. Among them, 20 miRNAs correlated with 18 predicted target genes that appear to be involved in pulmonary artery remodeling, mainly in three broad physiological processes: the hypoxia sensing response (HIF1a, NHE1, STAT5 and STAT3), endothelial permeability dysfunction (CD44, TRAF2, CDK2AP1, LZTFL1, JAZF1, PEBP1, LRP1B, RPS14 and THBS2) and inflammation (MEOX2, STAT5, STAT3, IRF8, MAP3K8, IL-1BETA and TNFRSF1B). Pathological pulmonary artery remodeling in the AS broilers was consistently observed in the present study. Taken together, the current analysis further illuminates the molecular mechanism of pulmonary artery remodeling underlying AS progression.

## INTRODUCTION

MicroRNAs (miRNAs) are a member of small noncoding RNAs family (21-23 nucleotides) which could target mRNA 3’- untranslated region and then at the post-transcriptional level to specifically silence a host of protein-coding genes [1]. Previous studies presented that miRNAs play a vital role in various biology aspects such as developmental timing, differentiation, cell death, proliferation, and metabolism [1, 2]. Newly, miRNAs dysregulation have been demonstrated to involve in a lot of cardiovascular diseases, including pulmonary hypertension (PH) [3]. Studies reported that miRNAs are the pivotal factors in regulating basic biological processes of PH, which include pulmonary artery smooth muscle cells (PASMCs) differentiation, proliferation and metabolism [4]. Typically, PASMCs in patients with chronic thromboembolic pulmonary hypertension (CTPH) have an exceptional miRNA profile and decreased expression of miR-let-7d, which is capable of promoting PASMC proliferation and may contribute to the development of CTPH [1]. According to the existing report, miR-17-92 overexpression downregulated the bone morphogenetic protein receptor type II [5], which has been often recognized as a crucial regulator in the pathogenesis of PH. Pullamsetti et al. have reported that miR-17 inhibition alleviated the vascular remodeling as well as the right ventricular hypertrophy in mouse with PH, and the over expressed miR-17 caused an increased proliferation of smooth muscle cells in human [6]. It was also demonstrated the down-regulation of miR-30c caused the up-regulated expression of platelet-derived growth factor receptor-β (PDGFRβ), and then activated the PDGF signaling which leaded to the PASMCs proliferation and phenotypes switching in a hypoxia condition [7]. Evidences indicated the miRNAs are crucial regulators in PH induced by hypoxia, and miR-190, miR-328 and miR-138 all help to promote pulmonary vascular remodeling [8–10]. Therefore, it is important to understand miRNA expression patterns for designing novel therapeutic strategies in broiler ascites syndrome.

Broiler ascites syndrome (AS), also called pulmonary artery hypertension (PAH) or pulmonary hypertension (PH), is a metabolic disorder that has been observed worldwide in fast growing broilers. Many researchers believe that AS related to nutritional, management, environmental and genetic factors [11–13] causes hypoxia in chickens, resulting in a series of pathophysiological changes including high pulmonary arterial blood pressure, pulmonary arterial remodeling, right ventricular hypertrophy and failure, the production of free radicals and increased lipid peroxidation [14]. Recent researchers found that the impact of pulmonary vascular remodeling on PAH progress refers to a complicated and multiple factorial process including various vasoactive molecules derived from endothelium like endothelin-1, insulin-like growth factors-II, vascular endothelial growth factor, transforming growth factor-a, PDGFR, erythropoietin, and hypoxia inducible factor-la (HIF-lα) [14–17]. These molecules have been recognized as pivotal regulators and possible therapeutic targets in treating PAH. It was reported protein-coding genes include multiple non-conserved sites and at least one conserved miRNA-binding site, and most of protein-coding genes may be under the control of miRNAs [18, 19].

Currently, the central role of miRNAs in the pulmonary arterial remodeling of AS is still not understood and changed miRNAs expression has been detected in some PH types. Here, we studied previously unknown miRNAs in pulmonary vascular remodeling and their target genes, and we also comment on the infusive miRNA-based therapies in the treatment of AS. We aimed to understand the regulation mechanism of miRNAs and their targeted genes in altering the remodeling phenotype in the pulmonary vasculature to provide candidate therapeutic miRNAs that can control the pulmonary artery remodeling underlying PH in both broiler chickens and humans.

## RESULTS

### Analysis of miRNA libraries built by small-RNA sequencing

**Figure 1:**
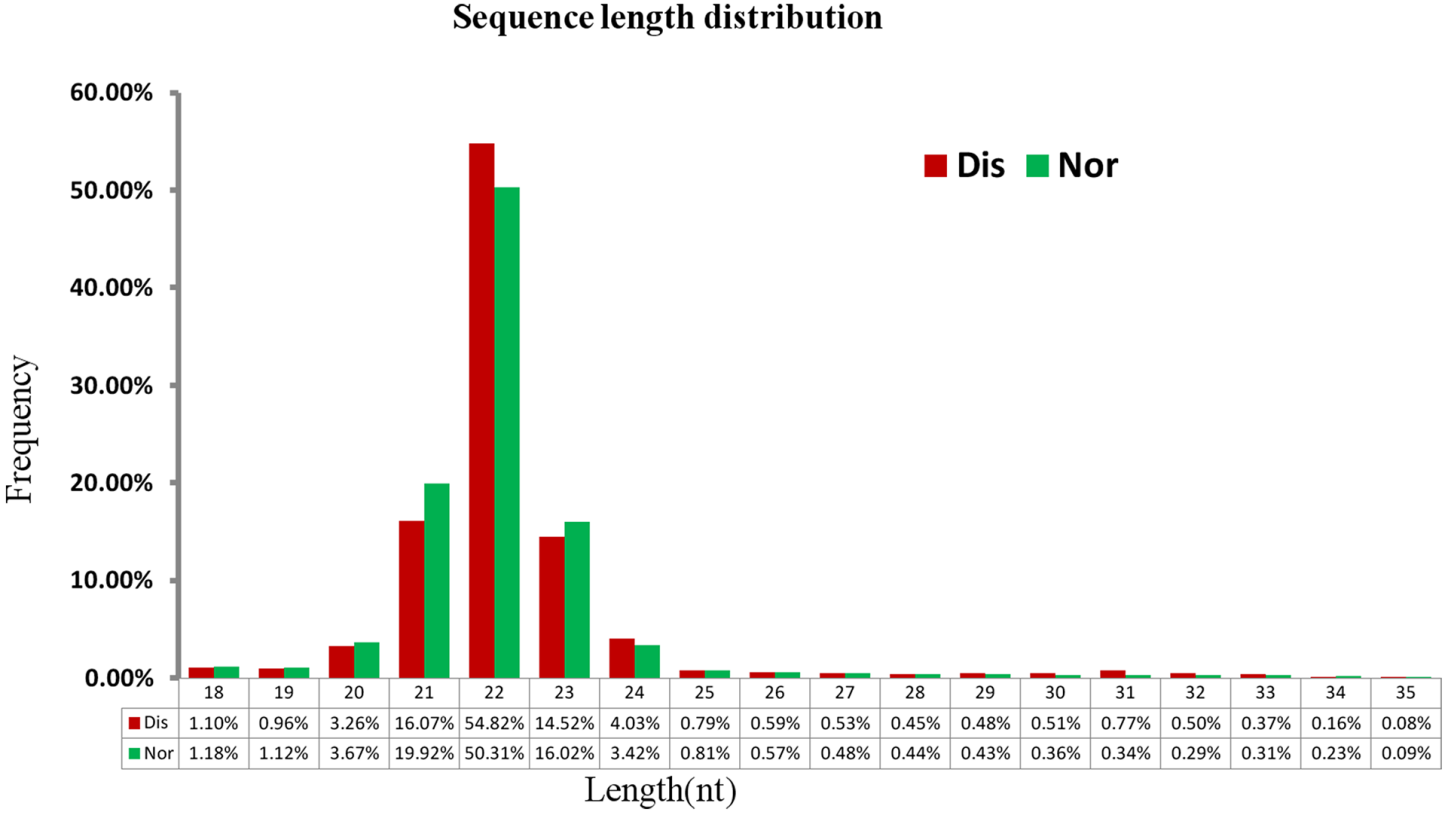
Length of small RNAs in the Nos and Dis libraries of broiler chickens X-axis, length of sRNA distribution; Y-axis, percentage of raw reads.

After pooling an equal amount of RNA from the pulmonary artery tissue of two broilers per group, we built and sequenced two libraries, the Nor and Dis sRNA libraries. As presented in the Table 1, we got 24,750,795 raw reads in total, of which 12,035,636 and 12,715,159 were separately produced in the Nor and Dis sRNA libraries. Raw reads of low quality only accounted for 0.02% of data in both the Nor and Dis libraries. After the adaptors, reads containing low-quality and poly-N tags were filtered out, a total of 11,673,403 (96.99%) and 12,538,289 (98.61%) clean reads were obtained from Dis and Nor pulmonary artery tissues. We selected the 18 to 35 nt long sequences from clean reads, with a high frequency in 21-23 nt size (Figure 1). The frequency of Nor sRNA was 50.31% (22 nt), then 19.92% (21 nt), 16.02% (23 nt), 3.67% (20 nt), and 3.42% (20 nt), whereas in the Dis sRNA library the frequency was 54.83% (22 nt) followed by 16.07% (21 nt), 14.52% (23 nt), 4.03% (24 nt) and 3.26% (20 nt). The percentage of 22 nt in length small RNAs was the highest among all sRNAs. A total of 10,262,122 (91.89%) and 10,996,505 (91.06%) unique small RNA reads were separately mapped to Nor and Dis this two libraries. The mapped small RNAs contain various RNA types which are shown in the Table 2.

**Table 1:** Statistical summary of the data generated by high-throughput small-RNA sequencing of broiler chicken pulmonary vessels

Read type	Sample		Total
	Nor	Dis	
total reads	12035636 (100.00%)	12715159(100.00%)	24750795
N% > 10%	3832 (0.03%)	4130 (0.03%)	7962
low quality	2787 (0.02%)	2398 (0.02%)	5185
5 adapter contamine	394 (0.00%)	376 (0.00%)	770
3 adapter null or insert null	344288 (2.86%)	163562 (1.29%)	163562
with ployA/T/G/C	10932 (0.09%)	10932 (0.09%)	21864
clean reads	11673403 (96.99%)	12538289 (98.61%)	24211692

total reads: the total number of raw reads; N% > 10%: The ratio of filtered out reads and the total raw reads, N content over 10%; low quality: The ratio of filtered out low quality reads and the total raw reads; 5_adapter_contamined: The ratio of 5_adapter_contamined reads and the total raw reads; 3_adapter_null or insert null: The ratio of filtered out 3_adapter_null or insert null reads and the total raw reads; with poly-A/T/G/C: The ratio of filtered out with poly-A/T/G/C reads and the total raw reads; clean reads: The ratio of clean reads and the total raw reads.

**Table 2:** Number of reads for each small RNA classification as identified in broiler chickens

Read type	sRNA libraries		Total
	Nor	Dis	
Total	10262122 (100.00%)	10996505 (100.00%)	21258627
Known miRNAs	3291711 (32.08%)	3377058(30.71%)	6668769
rRNAs	78426 (0.76%)	70244(0.64%)	148670
tRNAs	1177 (0.00%)	1005(0.01%)	2182
snRNAs	4997 (0.05%)	4584(0.04%)	9581
snoRNA	22863 (0.22%)	22260(0.20%)	45123
repeat	2870 (0.03%)	2617(0.02%)	5487
Novel miRNAs	107 (0.00%)	137(0.00%)	244
intron:+	67552(0.66%)	108526(0.99%)	176078
intron:-	10215(0.10%)	9526(0.09%)	19741
other	4939753(48.14%)	5924345(53.87 %)	10864098

Known miRNAs: perfectly matched mature miRNA sequences in the miRBase miRNA repository; Novel miRNAs: new mature miRNA sequences that were detected by characteristic stem-loop hairpins and minimum free energy (MFE); rRNAs, tRNAs, snRNAs, and snoRNAs are non-coding sRNAs; The miRNA sequences that were exon: + /exon:-/ intron: +/ intron:-, of the number of positive and negative chains and proportions; other: no known information is available.

### Identification of known miRNAs and putative novel miRNAs in broilers’ pulmonary artery tissues

In the current experiment, a total of 424 known mature miRNAs was found by using miRBase; of which, 362 were found in the Nor samples, and 369 were found in the Dis samples. The modified mirdeep2 and srna-tools-cli also predicted 23 novel miRNAs, with 18 and 19 found in the Nor and Dis samples, respectively (Additional file 1: Table S1).

### Characterization of differentially expressed miRNAs

**Figure 2:**
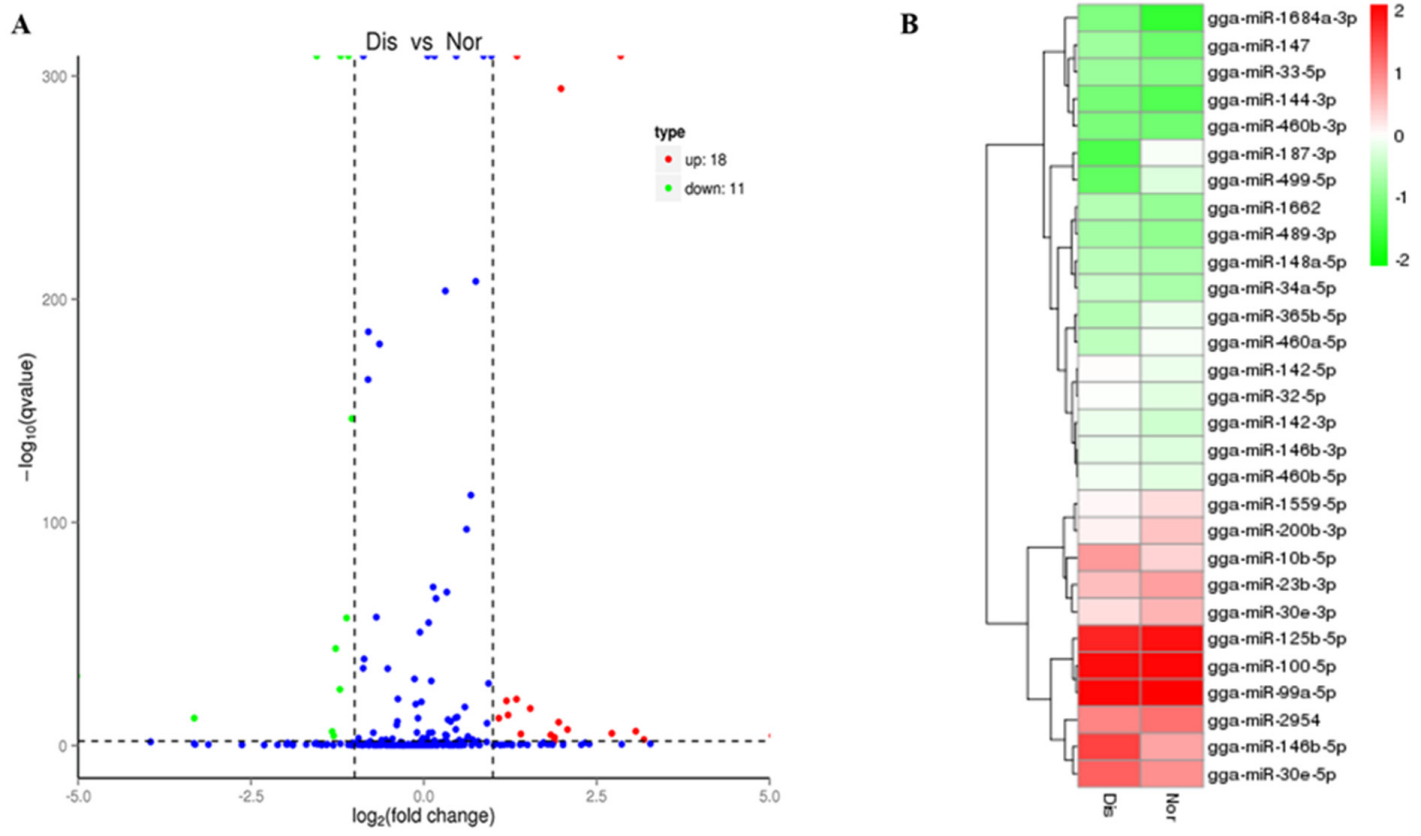
**A.** Volcano diagram displaying differentially expressed miRNAs between the Dis and Nor samples. The remarkably up-regulated miRNAs are shown in red, and down-regulated miRNAs are shown in green, whereas no difference is marked in blue with respect to the threshold of qvalue < 0.01 and |log2 (fold change) | > 1. **B.** Analysis of miRNAs expression clustering of Nor and Dis sample. Red indicates the high expression levels, blue indicates low expression.

To identify the key miRNAs regulating pulmonary artery remodeling underlying the AS process, the differentially expressed miRNAs in normal and diseased pulmonary arteries were compared. With the threshold of the qvalue < 0.01 and |log2(fold change)| > 1, 29 differentially expressed miRNAs were found both in known and novel miRNAs; of which, 18 were up-regulated, and 11 were down-regulated (Figure 2A) (Table 3). The heat map (Figure 2B) also presents variable expression patterns of those differentially expressed miRNAs in diseased and normal pulmonary arteries.

**Table 3:** Description of 29 significantly differentially expressed miRNAs

sRNA	Dis(read count)	Nor(read count )	log2.Foldchange	q.value	Regulation
gga-miR-146b-5p	14182.73	1970.06007	2.8478	0	Up
gga-miR-146b-3p	176.1897	75.7074039	1.2186	2.00E-14	Up
gga-miR-30e-5p	7595.017	2984.27247	1.3477	0	Up
gga-miR-10b-5p	2191.057	553.631236	1.9846	4.57E-295	Up
gga-miR-32-5p	239.7689	94.7176331	1.3399	1.62E-21	Up
gga-miR-142-5p	265.0928	115.728939	1.1957	8.95E-21	Up
gga-miR-142-3p	161.9114	55.6966364	1.5395	2.44E-17	Up
gga-miR-460b-5p	181.039	85.3792749	1.0843	5.49E-13	Up
gga-miR-460b-3p	14.81718	4.00215351	1.8884	0.0089826	Up
gga-miR-34a-5p	77.31873	20.0107676	1.95	3.48E-11	Up
gga-miR-1662	49.3008	11.6729477	2.0784	6.58E-08	Up
gga-miR-147	30.71197	3.66864072	3.0655	3.82E-07	Up
gga-miR-155	28.55675	4.3356663	2.7195	3.56E-06	Up
gga-miR-148a-5p	54.68886	20.6777931	1.4032	6.76E-06	Up
gga-miR-489-3p	36.90825	10.3388966	1.8359	1.70E-05	Up
gga-miR-1684a-3p	16.4336	0	5.0386	3.52E-05	Up
gga-miR-33-5p	27.20973	7.33728144	1.8908	0.00021662	Up
gga-miR-144-3p	12.12315	1.33405117	3.1839	0.0020304	Up
gga-miR-365b-5p	47.14557	116.395965	-1.3038	4.30E-05	Down
gga-miR-2954	3266.514	6720.61628	-1.0408	3.14E-147	Down
gga-miR-23b-3p	1028.582	2230.86707	-1.1169	6.44E-58	Down
gga-miR-30e-3p	525.3363	1271.01725	-1.2747	3.40E-44	Down
gga-miR-187-3p	4.579855	148.746705	-5.0214	6.84E-32	Down
gga-miR-200b-3p	350.763	813.437701	-1.2135	6.64E-26	Down
gga-miR-100-5p	48124.58	102433.452	-1.0898	0	Down
gga-miR-125b-5p	29025.51	84941.7061	-1.5492	0	Down
gga-miR-499-5p	7.812694	78.0419935	-3.3204	4.99E-13	Down
gga-miR-460a-5p	65.46499	164.088294	-1.3257	5.40E-07	Down
gga-miR-99a-5p	55817.12	128480.467	-1.2028	0	Down

The q-value indicates the adjusted–P value.

### Target gene prediction and functional annotation

**Figure 3:**
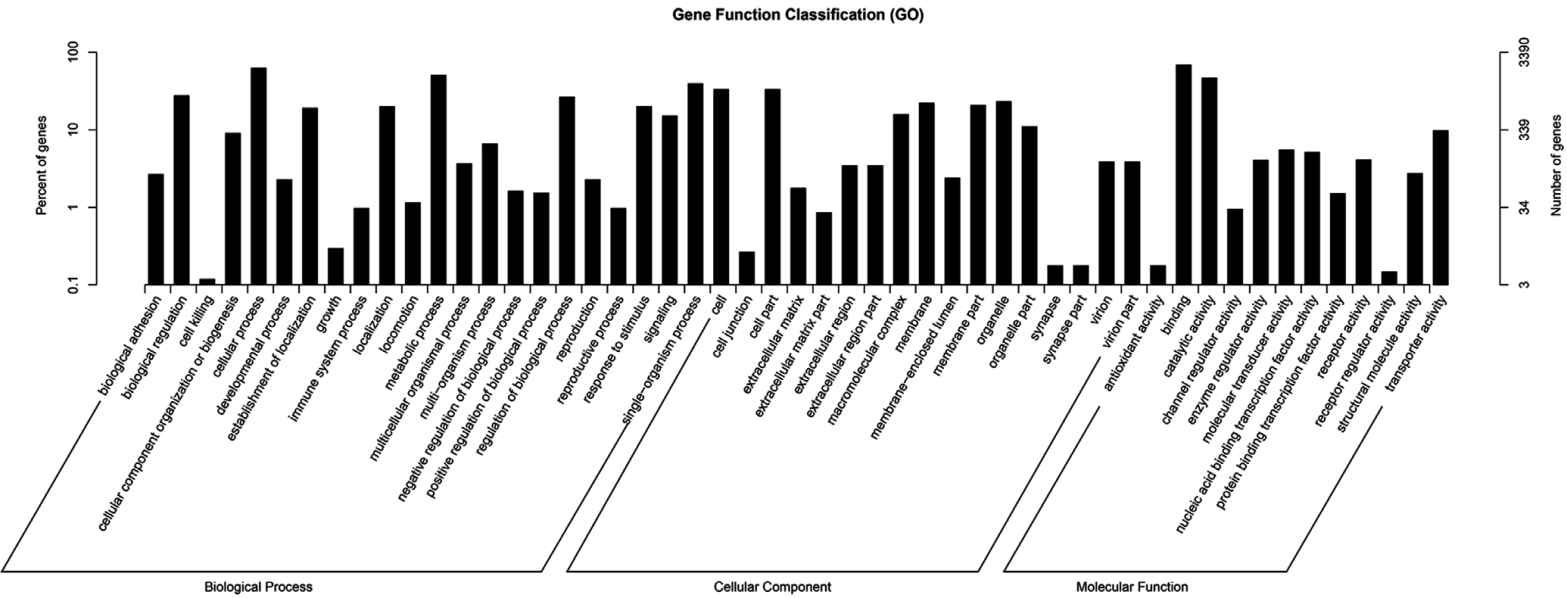
GO functional enrichment analysis for predicted target genes of the 29 differentially expressed miRNAs in pulmonary artery tissues from the Dis and Nor broilers The right-hand-side scale indicates the targeted gene numbers corresponding to the GO terms; the left-hand-side scale indicates the percent of targeted gene numbers corresponding to the GO terms.

The target genes of miRNAs are effectively searched based on the sequence complementarity among miRNAs and their relevant target genes [20]. In this study, 5497 target genes in total were identified for 29 significantly differently expressed miRNAs (Additional file 2: table S2). Each differential expressed miRNAs has various target genes ranging from one to more than one hundred. GO is an international standardized gene functional classification system to comprehensively describe the characteristics of those targeted genes of differentially expressed miRNAs. Figure 3 shows biological process, cellular component and molecular function these three main GO categories with 51 total subcategories. In the category of biological process, the dominant subcategories were ‘biological process’ (1,816, 53.57%), followed by ‘cellular component’ (441, 13.01%) and ‘molecular function’ (1,050, 30.97%). On the other hand, these identified miRNA target genes were also involved in total 153 biochemical KEGG pathways. “Metabolic pathways” was the most significantly enriched one with regard to the rich factor and gene number (319 genes), followed by “focal adhesion” (61 genes), “protein processing in endoplasmic reticulum” (50 genes), “RNA transport” (47 genes) and “ubiquitin mediated proteolysis” (44 genes). Additional file 3: Table S3 displayed these KEGG pathways’ details including id names, gene names and hyperlinks. These annotations of GO and KEGG enrichment analysis offer a worthy resource for further detecting specific processes and pathways that are involved in the process of ascites syndrome in broilers.

### Association analysis of miRNA-mRNA

To better define the corresponding relationship among miRNAs and genes which were both differential expressed in pulmonary artery tissues from normal and AS broilers, we used a combined analysis of miRNA and mRNA in the same samples. The 29 differentially expressed miRNAs and their predicted target genes are shown in Additional file 4: table S4; each miRNA regulates no more than one target gene. We previously also performed mRNA sequencing analysis in the same pulmonary artery tissue samples used to perform miRNA analysis, and 895 differentially expressed genes in total were found between the pulmonary artery tissues from normal and AS broilers [21]. In this study, these total 895 differentially expressed genes were added to the candidate miRNAs that can regulate them, and those genes have a number of regulatory miRNAs ranging from zero to dozens (Additional file 5: table S5). A number of genes, which are differentially expressed and are also the targets of miRNAs that are differentially expressed in the pulmonary arteries of AS broilers as detected by RNA-sequencing, were selected for GO and KEGG analysis. Additional file 6: Figure S1 and Additional file 7: Figure S2 display different functional categories of those targets, which indicate the molecular and cellular events involved in pulmonary artery remodeling development underlying AS development.

**Figure 4:**
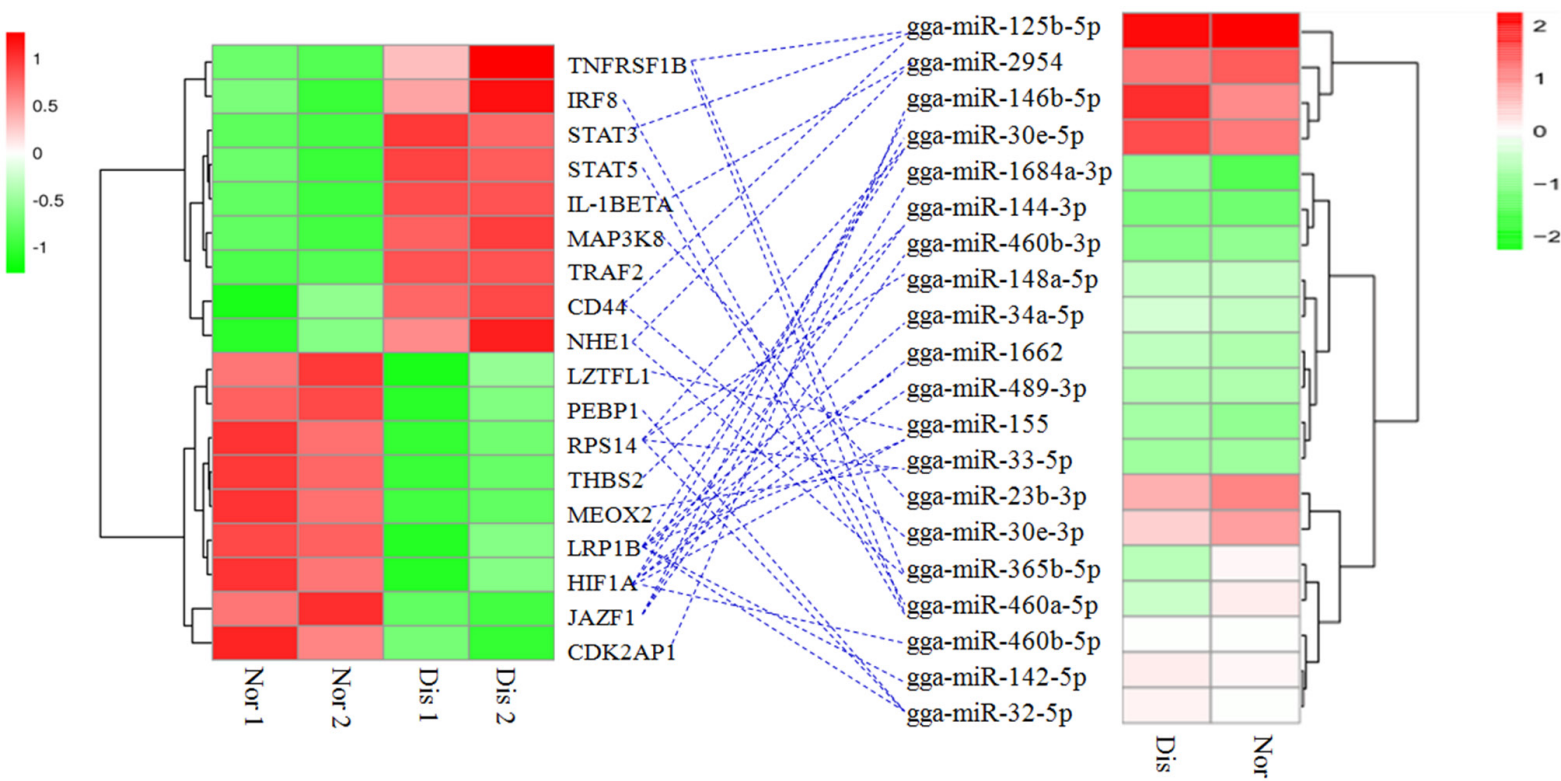
Association of miRNA-mRNA The left heat map indicates the 18 differentially expressed genes, and the right heat map indicates the 20 differentially expressed miRNAs in AS and normal broiler’ pulmonary artery tissues. The blue dotted line indicates the miRNAs correlated with the corresponding target genes. Nor 1 and Nor 2 indicate the replicates of the pulmonary artery tissues of the normal broilers, and Dis 1 and Dis 2 indicate the replicates of the pulmonary artery tissues of the diseased broilers.

After trimming, it was found that, except for gga-miR-147, 28 other differentially expressed miRNAs have 162 targeted genes, which were also differentially expressed in pulmonary arteries by the RNA-sequencing method. Among those differential miRNAs and related targets, we noticed 20 miRNAs correlated with 18 differentially expressed target genes that are closely related to pulmonary artery remodeling in the progress of AS, mainly through three broad physiological processes (Figure 4): the hypoxia sensing response (HIF1α, NHE1, STAT5 and STAT3), endothelial permeability dysfunction (CD44, TRAF2, CDK2AP1, LZTFL1, JAZF1, PEBP1, LRP1B, RPS14 and THBS2) and inflammation (MEOX2, STAT5, STAT3, IRF8, MAP3K8, IL-1BETA and TNFRSF1B).

### Validation of miRNAs expression by qRT-PCR

**Figure 5:**
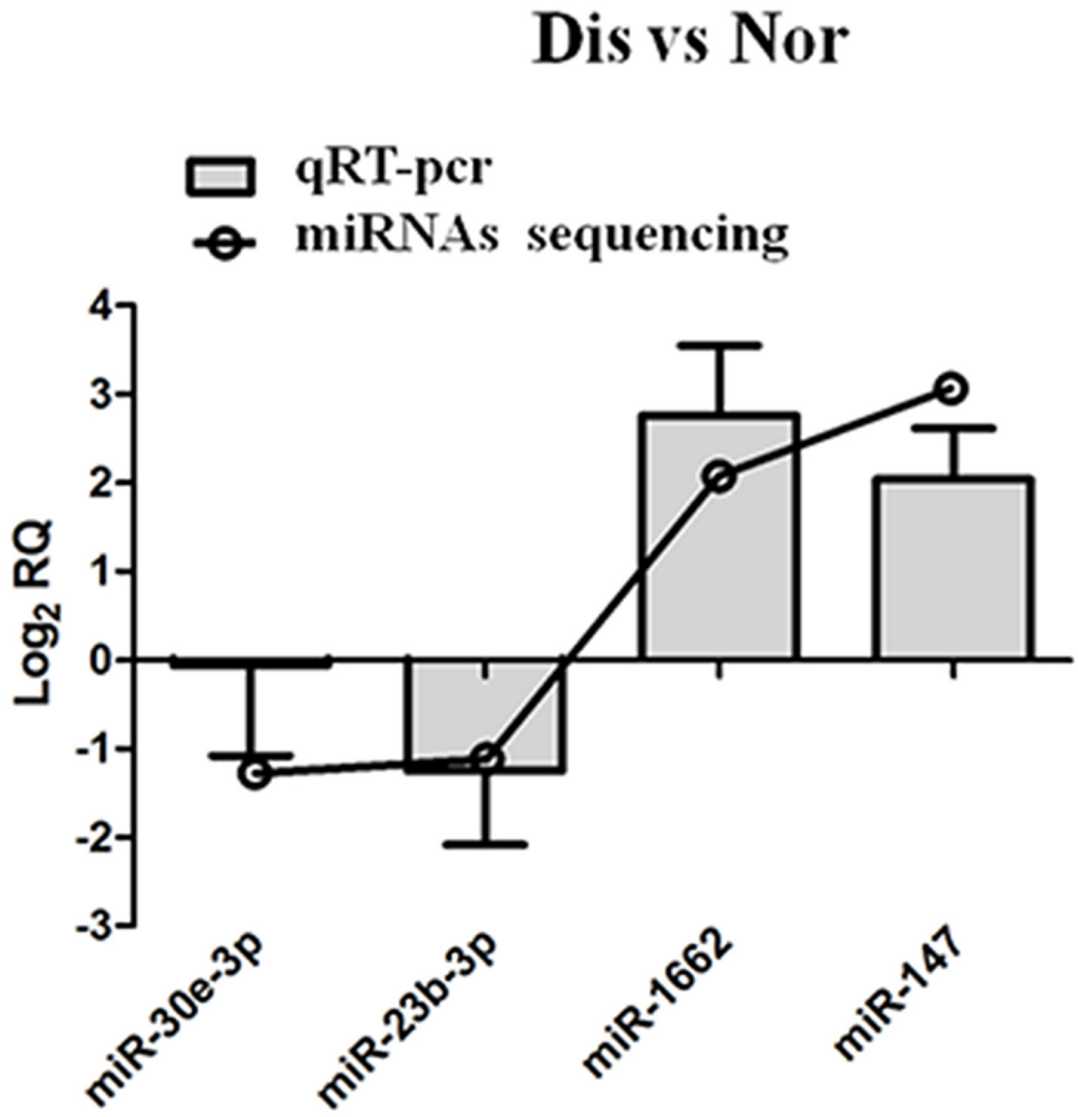
qRT-PCR validation of miRNAs in pulmonary artery tissues from Dis and Nor broilers

To validate the present results of miRNA-sequencing, miR-30E-3P, miR-187-3P, miR-1662, and miR-147 these four miRNAs were selected from all miRNAs identified in this study for qRT-PCR validation. The confirmation results in Figure 5 indicate that the expression pattern of four miRNAs by qRT-PCR is consistent with the miRNA-sequencing.

### Pathological changes in the pulmonary artery

**Figure 6:**
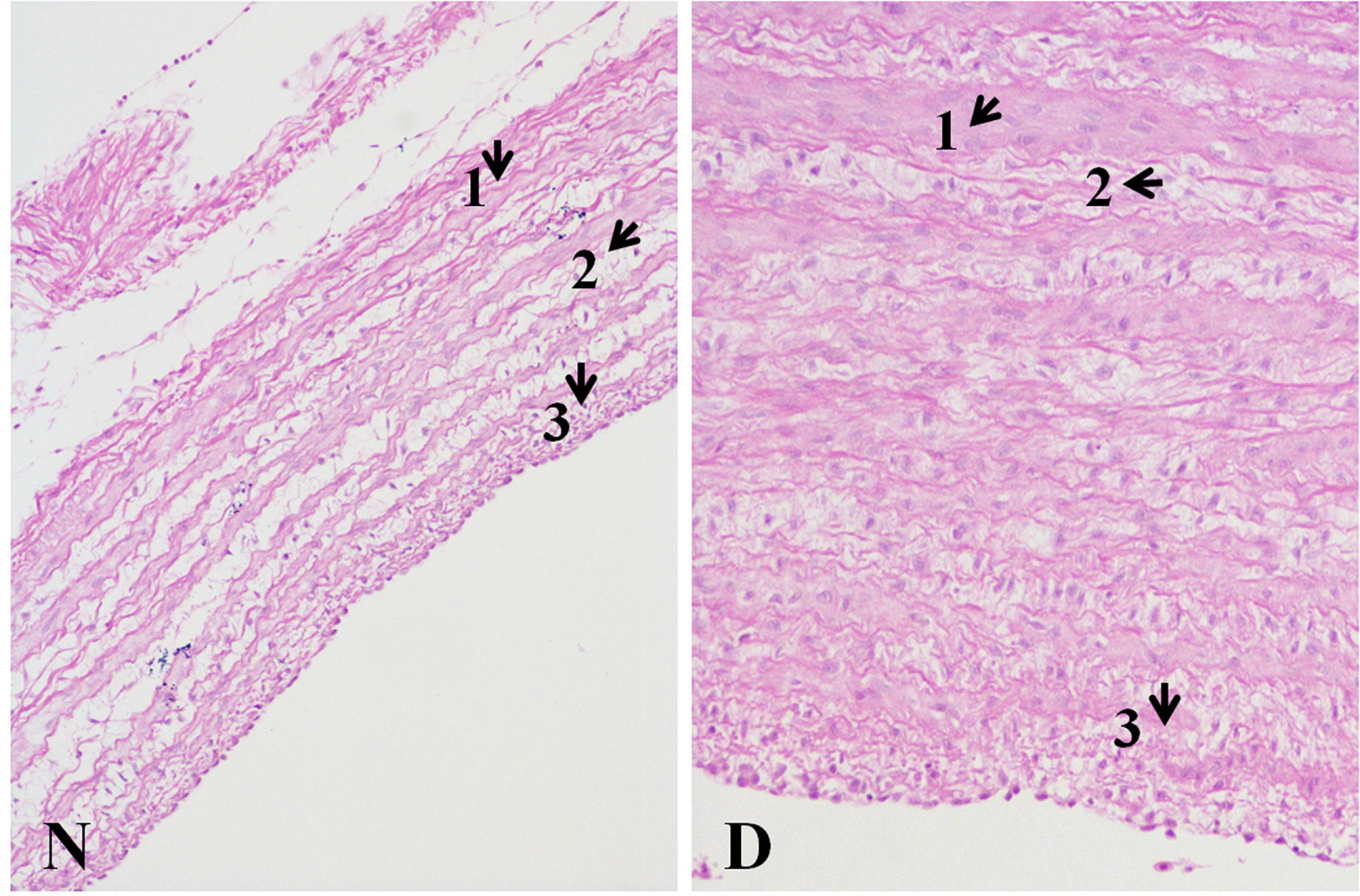
Histopathology changes of pulmonary artery tissues (200X, HE) The wall thickness of the Dis pulmonary artery sample was markedly thickening Comparing to the N tissue sample, in the D tissue sample, 1) the smooth muscle fiber is thicker; 2) excess fiber production was found; and 3) a more compact intima was observed. The histopathological changes in the picture were pointed by arrows.

Figure 6 displays histopathology changes of pulmonary artery from broilers. The pulmonary artery thickness from the AS broilers was significantly increased, and the medial smooth muscle layers were thicker and were discontinuous and disordered. Moreover, the mesenchyme between muscle layers became looser in the AS broilers than that in the normal broilers.

## DISCUSSION

Many studies proved the miRNAs expression patterns are tissue-specific and cell-specific and may become dysregulated during various disease processes, and a wild range of miRNA expression levels were reported to correlate with disease severity [22]. Small RNA sequencing can reveal miRNAs expression at global level; therefore, it has become a helpful tool to identify some functional miRNAs. Date proved that a lot of miRNAs have been related to pulmonary arterial hypertension [10, 23]. In this present experiment, we demonstrated the miRNAs expression profiles of pulmonary arterial remodeling from broilers with and without AS. The sequencing results indicated that small RNAs in Nor and Dis broiler pulmonary arterial samples had a major size range in 18-35 nt, and the 22 nt is dominant. This result accords with the already reported 19-24 nt range for miRNAs, and previous researches in chicken breast muscle tissues, skeletal muscle and ovary revealed alike results [24–26].

Pulmonary arterial remodeling is a presently irreversible pathological hallmark of PAH [14]. This complex disease involves the pathogenic dysregulation of all cell types within the small pulmonary arteries contributing to vascular remodeling leading to intimal lesions, resulting in elevated pulmonary vascular resistance and right heart dysfunction [14, 20]. Based on the current sequencing results, we found 447 miRNAs, containing 424 known miRNAs and 23 novel miRNAs. And 29 differentially expressed miRNAs were found both in known and novel miRNA categories; 18 miRNAs were up-regulated, and 11 miRNAs were down-regulated (Table 3). It is implicated that miRNAs is involved in the PAH process by regulating cell biological processes like proliferation, and what has been demonstrated is that resistance to apoptosis and excess proliferation of PASMCs are the main characteristics of PAH [7]. And Caruso et al firstly reported miRNAs dysregulation in the progress of PAH by the means of microarray analysis and quantitative polymerase chain reaction, who detected that miR-451and miR-322 up regulated, whereas miR-30, miR-22, and let-7f down regulated in PAH rodent models [27]. This research highlighted the potential role of specific miRNAs in disease progress [23], like miR-21, miR-204, miR-17, miR-155, miR-138 and miR-30c in human and rat models of PAH [7, 8, 28, 29]. In this study, we found that miR-155, miR-23b-3p, miR-146b-5p and miR-146b-3p upregulated in AS broilers’ pulmonary arteries tissue.

MiR-155 was among the miRNAs that were regulated in endothelial cells by shear stress forces [30, 31]. MiR-155 is expressed in multiple cell types, and it has been implicated in activating several biological processes including hematopoiesis, inflammation and immunity [32]. Increased miR-155 levels in human Endothelial Cells (ECs) induced changes in morphology and filamentous-actin organization [33]. In addition, the pattern of differential expression for miR-23b-3p was reported to contribute to the proangiogenic response [20]. The predicted functions of mir-23b-3p showed significant associations with the migration of phagocytes, the proliferation of mononuclear leukocytes and the cell movement of smooth muscle cells [20]. The miR-146b and miR-146a these two members of miR-146 family, which negatively regulate expressions of inflammatory genes in some cell types containing airway smooth muscle, endothelial, epithelial cells and fibroblasts, are upregulated in a murine asthma model [34]. Considering the differential expressions of miR-155, miR-23b-3p, miR-146b-5p and miR-146b-3p found in the AS broiler pulmonary artery tissues in this study, we proposed these four miRNAs may also serve as a key regulator to pulmonary artery remodeling during AS progress. Nonetheless, miR-142-3p, miR-449-5p, miR-148a-5p, miR-365b-5p, miR-200-3p, miR-34a-5p and miR-1443-3p found in our results have few reported associations to PAH. Therefore, these differentially expressed miRNAs might play an important role in chickens with PAH and may also lay a foundation for identifying novel target therapeutic miRNAs to regulate PAH in humans.

### MicroRNAs-mRNA association analysis

Many factors contribute to gene expressions at translational level and transcriptional level during disease progress. MiRNAs with 22 to 25 nucleotides, are a class of small noncoding RNA molecules and function in regulating gene expressions at post-transcriptional level. MiRNAs can downregulate the expression of mRNA targeted genes by partially binding mRNAs complementary sequences and then degenerating and/or inhibiting their translations [35]. In our results, miRNA analysis and miRNA-mRNA association analysis found 29 miRNAs that regulate a total of 162 significant differentially genes in AS broilers’ pulmonary artery tissues. We noticed some miRNAs correlated with target genes that appear to be involved in the pulmonary artery remodeling, mainly through three broad physiological processes (Figure 4): the hypoxia sensing response (HIF1α, NHE1, STAT5 and STAT3), endothelial permeability dysfunction (CD44, TRAF2, CDK2AP1, LZTFL1, JAZF1, PEBP1, LRP1B, RPS14 and THBS2) and inflammation (MEOX2, STAT5, STAT3, IRF8, MAP3K8, IL-1BETA and TNFRSF1B).

### Early response to hypoxia

Hypoxia is a noted cause of pulmonary vascular remodeling during PH [7]. We observed that multiple differentially expressed microRNAs correlated with the downregulated HIF1α and the upregulated NHE1, STAT5 and STAT3 in pulmonary arteries from AS broilers compared to the controls. HIF1α is a critical downstream mediator for HIF, which is a pivotal regulator for a wide range of genes and pathways to adapt to hypoxia [36]. Previous research has found that the partial deficiency of HIF1α in heterozygous mice reduced the degree of pulmonary artery remodeling in comparison with normal mice when in a hypoxic condition [23]. Furthermore, many miRNAs has been demonstrated to be dysregulation when in a hypoxic condition, and this dysregulation are in a HIF-dependent way [23]. It has been confirmed that the hypoxia inducible miR-155 were upregulated in hypoxia and reduced its target gene HIF1α expression both in mRNA and protein levels [36]. And it was demonstrated that miR-155 may serve as a component of a negative-feedback loop which specifically controls HIF1α resolution and finally determines transcriptional response in cells when in exposer to sustained hypoxia [36]. In our results, a similar observation was found: that up-regulated miR-155 also correlated to HIF1α, which was under expressed in the pulmonary arteries of AS broilers. We also identified five other microRNAs including miR-489-3p, miR-1662, miR-460b-5p, miR-144-3p and miR-30e-5p, which were involved in the regulation of HIF1α in these data. Overexpression of NHE1 was found in mice with vascular remodeling characteristic in PH progression induced by hypoxia [37]. Based on the present data, both down-regulated miR-365b-5p and miR-2954 correlated with NHE1 expression. A previous study reported that the deficiency of NHE1 prevented the vascular remodeling and occurrence of PH in mice exposer to hypoxia, together with the PASMCs proliferation being highly reduced and thickness of pulmonary arteries’ medial wall being decreased [37]. In this study, we also found that the down-regulated miR-460a-5p and miR-125b-5p were relevant to STAT3 and STAT5 these two genes, respectively. In fact, other research has reported that STAT3 and STAT5 expressions were up-regulated in the lung tissue of rat models with hypoxic pulmonary hypertension (HPH), suggesting that the STATs are involved in the pathogenesis of HPH formation [38].

### Endothelial cell dysfunction

It was shown that endothelial cell dysfunction promoted the pulmonary arterioles remolding which contributes to PH occurrence [14]. We found that the upregulation of CD44 and downregulations of CDK2AP1, LZTFL1, JAZF1, PEBP1, LRP1B and THBS2, were associated with some microRNAs (Figure 4). CD44, is one kind of transmembrane glycoprotein, which was found to be involved in various cellular functions containing hematopoiesis, cell migration and tumor metastasis [39]. It was reported that an upregulation of CD44 leads to the development of inflammation and angiogenesis in retinas [40]. From the current association analysis data, we observed that both differentially expressed miR-30e-3p and miR-125b-5p were associated with overexpression of CD44 in the pulmonary arteries of AS broilers. LRP1B, belonging to the low-density lipoprotein receptor family, was recognized as an important tumor suppressor, and LRP1B under-expression was found in various primary cancers. Prazeres et al. observed that overexpression of miR-548a-5p markedly paralleled decreased LRP1B expression in thyroid cancer cells [41]. And in this study, we found more than one miRNA, including miR-142-5p, miR-32-5p, miR-1662, miR-155, miR-30e-5p and miR-34a-5p, that function together to target LRP1B in the pulmonary arteries of AS broilers, which was characterized by vascular cells proliferation. CDK2AP1 was shown to play a role in epithelial-to-mesenchymal transition, determining both cell fate and differentiation [42]. Significant upregulation of miR-146b-5p and its corresponding downregulation of CDK2AP1 were observed in the AS pulmonary arteries in our findings, which is consistent with Zhong et al., who reported that significant upregulation of miR-205 targeted the downregulation of CDK2AP1 in the laryngeal squamous cell carcinoma [43]. Likewise, from the miRNA-mRNA association, the under expressed genes LZTFL1, JAZF1, THBS2 and RPS14 were associated with microRNAs (miR-146b-5p, miR-1684a-3p, miR-460b-3p, miR-30e-5p, miR-33-5p, miR-148a-5p, miR-32-5p, miR-155 and miR-144-3p) that were down-regulated in pulmonary arteries (Figure 4). LZTFL1, expression in epithelial cells of various tissues such as the lung, is down-regulated in some tumors [44]. Overexpression of JAZF1 protected against the development of atherosclerosis in mice [45]. Mice that lacks THBS2 developed a complex phenotype characterized by abnormalities in connective tissues and fibroblast adhesion, elevated endosteal bone growth and an increase in vascular density [46]. Ribosomes have been presented to be related to cell proliferation, growth and apoptosis, and low expression of RPS14 had been found in myelodysplastic syndrome patients [47]. Our results implicate that these miRNAs changes will result in changes of vessel wall and function as a barrier to pulmonary arteries further leading to AS occurrence by affecting some target genes at the post transcriptome level.

### Inflammation

Inflammation contributes to the pathophysiology of cardiovascular disease, and in both humans and broilers, inflammation serves as a contributor to pulmonary artery remodeling, which activates the development of PH [14]. Through a miRNA-mRNA association analysis (Figure 4), we identified several differentially expressed miRNAs that regulated some important target genes including IRF8, MEOX2, IL-1BETA, TRAF2, TNFRSF1B and MAP3K8, which are associated with inflammation. IRF8, firstly identified in immune cells, controls the development and differentiation of endothelial cells and vascular smooth muscle cells [48]. IRF8 was also confirmed to be vital in modulating the phenotypic switching of smooth muscle cells and neointima formation by directly interacting with myocardin complex when in answer to vascular injury [49]. Previous research has found that expression of miR-22 performed a significant effect on IRF8 mRNA abundance in development of dendritic cells [50]. Similarly, we found that down regulated miR-460a-5p also correlated with the overexpression target gene IRF8 in the AS broilers’ pulmonary artery tissues. We noticed the miR-33-5p, miR-460a-5p, miR-365b-5p, miR-125b-5p and miR-2954 correlated with inflammatory genes including MEOX2, IL-1BETA, TRAF2, TNFRSF1B and MAP3K8, and except for the up-regulated miR-33-5 correlated with under expression of MEOX2, other down-regulated miRNAs all correlated with overexpression targets. MEOX2 expression, maximal in quiescent ECs, inhibits EC proliferation and angiogenesis and NF-kB signaling in response to proangiogenic factors [51]. Our results showed that the up-expression of miR-33-5 was associated with the down-regulation of the target MEOX2, which may be conducive to pulmonary vascular growth and further pulmonary artery remodeling. TNFRSF1B was found to play a significant role in the initiation and progression of arteriosclerosis and promotes adaptive arteriogenesis and angiogenesis [52]. MAP3K8 forms a complex in the quiescent state and can be activated by pro-inflammatory stimuli, such as TNF-α, IL-1BETA and LPS [53]. TRAF2 is required for NF-κB activation in airway smooth muscle cells [54]. Altogether, these target genes of miRNAs found in this study may promote the pulmonary artery remodeling by being involved in inflammation and proliferation.

The histopathology examination in this study (Figure 6) observed the pulmonary arterial remodeling in AS broilers and it was characterized by the increased thickness of artery wall, particularly in the medial intima made up of vascular smooth muscle cells. Previous pathological research in AS also reported alike features of pulmonary artery remodeling, which is a result of cellular proliferation, hypertrophy and sparse migration [55]. The present histopathological changes further conformed that the current findings of differentially expressed miRNAs and their correlated targets may be main molecular regulators for pulmonary artery remodeling in AS progress.

In conclusion, we identified a total of 29 differential expressed miRNAs with 5497 predicted target genes in pulmonary artery tissues from broilers with and without AS. And miRNAs-mRNA association analysis identified that those 29 miRNAs regulate a total of 162 significant differentially target genes. It was suggested that dysregulated miRNAs and mRNAs may be involved in three broad physiological processes: hypoxia sensing response, endothelial cell dysfunction and inflammation, further developing pulmonary artery remodeling underlying AS progression.

## MATERIALS AND METHODS

### Ethics statement

All animal experiments were approved by the Institutional Animal Care and Use Committee of Jiangxi Agricultural University. All care and procedures to the broiler chickens were conducted in strict accordance with the guidelines of the Institutional Animal Care and Use Committee of Jiangxi Agricultural University. All efforts were made to minimize the suffering of the animals.

### Animals and sample collection

Seventy-five male Arbor Acre commercial broiler chickens (21 days old) were randomly divided into a normal group (Nor) (n = 20) and a diseased group (Dis) (n = 55). Until they were 21 days old, all birds were reared at a room temperature of 20-23°C. Starting from the age of 21 days, birds in the normal group were maintained at a room temperature of 20 -25 °C and provided with tap water, whereas the broilers in the disease group were maintained at a low room temperature (approximately 14°C) and given water with 0.3% salt to induce AS [56, 57]. All birds were allowed to have free access to the same diet ad libitum and had a 23h fluorescent illumination per day throughout the trial period. AS peak was at 35 days old in broilers, so we began to collect samples from the time points of 28 to 42 days. To obtain the AS positive birds in a timely manner, we paid close attention to the birds and observed whether the broilers had symptoms indicative of AS, such as reluctance to move, AS-like depression, open-beak breathing, a distended abdomen, or cyanosis. Once those symptoms were observed, the birds were immediately sampled. They were weighed, and a total of 2 mL of blood per broiler was taken from the brachial vein and deposited in a tube containing heparin for a routine blood examination using an Auto Hematology Analyzer (PE-6300 VET, China). After that, the pulmonary artery was immediately collected and quickly rinsed in saline water. A portion of the pulmonary artery was flash-frozen in liquid nitrogen and stored in a -80°C freezer for RNA-seq and microRNA analysis, whereas other parts of the pulmonary artery tissue were cut and stored in 10% formaldehyde for pathological observation. Finally, to measure the Ascites Heart index (AHI), we removed the atrium and aorta, and the right ventricle (RV) was separated from the septum and left ventricular; then, the RV (right ventricle) and TV (total ventricles) were weighed, and the ratio of RV/TV was calculated.

To select the positive AS broilers and obtain their pulmonary artery for RNA-seq and microRNA analysis, we collected the samples based on the judgment criteria of AS, including AHI > 0.28, HCT > 36% and the presence of yellow liquid in the abdominal cavity and pericardium [14]. Normal chickens were selected from the normal group (AHI < 0.219; HCT < 28% and free of yellow liquid in abdominal cavity and pericardium). Finally, we randomly chose two AS-positive broilers from the disease group and two normal broilers from the normal group and obtained their pulmonary artery tissues and extracted the miRNA for sequencing analysis. We used the same pulmonary artery tissue from the two positive-AS broilers and the two normal broilers’ for miRNA-sequencing analysis to perform RNA-sequencing, allowing for two replicates per group.

### Total RNA extraction, sRNA library construction and sequencing

The total RNAs per broiler's pulmonary artery were extracted using the miRNAeasy Mini Kit (Qiagen, Valencia, CA, USA) following the manufacturers’ protocols. For high throughput small RNA sequencing, we pooled equal amounts of the total RNA from two biological replicates per group. RNA degradation and contamination were evaluated using 1% agarose gels, and the RNA purity was checked using a NanoPhotometer® spectrophotometer (IMPLEN, CA, USA). All frozen RNAs were tested using a 2100 Bioanalyzer (Agilent Technologies, Santa Clara, CA, USA) for quality control (RNA quality index > 5.0). After that, 3 μg of total RNA per sample was used as the input material for the small RNA library preparation. Sequencing libraries were generated using the NEBNext® Multiplex Small RNA Library Prep Set for Illumina® (NEB, USA), and index codes were added to attribute sequences to each sample. For each sample, small RNAs (18-35 nt) were ligated with 5’ and 3’ RNA adapters by T4 RNA ligase, after which they were purified by electrophoretic separation on a 15% TBE-urea denaturing PAGE gel from total RNA. Library quality was assessed on the Agilent Bioanalyzer 2100 system using DNA High Sensitivity Chips. The clustering of the index-coded samples was performed on a cBot Cluster Generation System using a TruSeq SR Cluster Kit v3-cBot-HS (Illumina). After cluster generation, the library preparations were sequenced in the form of 50 bp single end reads on an Illumina HiSeq 2500 platform at the Novogene Company, Beijing, China.

### Data analysis

#### Quality control and reads mapping

Raw data (raw reads) generated via the Illumina HiSeq 2500 platform were processed through custom perl and python scripts, whereas clean data (clean reads) were obtained from the raw data by removing reads containing poly-Ns, with 5’ adapter contaminants, without a 3’ adapter or the insert tag, containing poly-A or T or G or C and/or low quality reads. At the same time, the Q20 scores, the Q30 scores and the GC-content of the raw data were calculated to control the library quality. We chose a certain length range from the clean data reads to perform all the downstream analyses. The small RNA tags without mismatches were mapped to reference sequences (Gallus gallus;ftp://ftp.ensembl.org/pub/release-76/fasta/gallus_gallus/dna/) by Bowtie (bowtie-0.12.9) to analyze their expression and distribution along the reference.

#### Known miRNA alignment and novel miRNA prediction

Mapped small RNA tags were used to search for known miRNAs with the miRBase 20.0 referencing software (http://www.mirbase.org/). The modified software programs mirdeep 2 and srna-tools-cli were used to generate the potential miRNAs and to draw their secondary structures. The available software miREvo and mirdeep2 were integrated to predict novel miRNA.

#### miRNA differential expression and target genes prediction analysis

In our analysis pipeline, known miRNAs were searched against the miFam.dat (http://www.mirbase.org/ftp.shtml) database to identify families; novel miRNA precursors were submitted to Rfam (http://rfam.sanger.ac.uk/search/) to look for Rfam families. Positions 2~8 of a mature miRNA were called the “seed region”, which was highly conserved. The target of a miRNA might be different with changes in nucleotides in this region. In the current analysis pipeline, miRNA, which might have base edits that could be detected by aligning all the sRNA tags to mature miRNAs, was allowed to have one mismatch. The miRNA expression levels were estimated by TPM (transcript per million) using the normalization formula (normalized expression = mapped read count/total reads*1000000) [58]. Differential expression analysis of pulmonary artery tissues from AS broilers and normal broilers was performed using the DEGseq (2010) R package. The qvalue is an adjusted P-value, and both qvalue < 0.01 and |log2(fold change)| > 1 were set as the threshold for significantly differential expression. Predicting the miRNA target gene was performed using psRobot_tar in miRanda for animals.

The data that were analyzed in this study are available in the SRA public repository with the accession number SRP068885.

#### GO and KEGG enrichment analysis

Gene Ontology (GO) enrichment analysis for the target gene candidates of differentially expressed miRNAs was based on the Wallenius non-central hyper-geometric distribution. KEGG (Kyoto Encyclopedia of Genes and Genomes), a database resource for understanding high-level functions and utilities of the biological system, was used to determine the functional diversity of all target genes predicted in those differentially expressed miRNAs (http://www.genome.jp/kegg/). KOBAS software was further used to test the statistical enrichment of the target gene candidates in KEGG pathways.

#### mRNA library construction, sequencing and identification of differentially expressed genes

We used the same two AS broilers and two normal broilers from the above birds that were used to perform the miRNA sequence analysis to obtain their pulmonary artery tissues to perform mRNA sequencing. This allowed for two replicates per group for the mRNA sequencing analysis in this study. Total RNA was extracted from the pulmonary artery using the TRIzol reagent (Invitrogen, Burlington, ON, Canada) according to the manufacturer's protocol. After measuring the RNA concentration and integrity, a total of 3 μg RNA per sample was used as an input for RNA sample preparation. We used the NEBNext® Ultra™ RNA Library Prep Kit for Illumina® (NEB, USA) to generate sequencing libraries. The library preparations were then sequenced on an Illumina HiSeq 2500 platform at the Novogene Company, Beijing, China, and 50 bp single-end reads were generated. After data analysis, the differentially expressed genes in the pulmonary arteries of the AS broilers and the normal broilers were analyzed.

#### Association analysis of miRNAs-mRNA

MiRNAs are a class of small noncoding RNA molecules that function in the post-transcriptional regulation of gene expression. In this study, differentially expressed miRNAs and their target genes were analyzed by miRNA-sequencing. We also sequenced the RNA from the same pulmonary artery samples to detect the differentially expressed genes underlying ascites syndrome, and the datasets are available in SRA public repository with the accession number SRP068247. To determine whether the important differentially expressed miRNAs’ predicted target genes are also differentially expressed, we used the perl language program to extrapolate the mRNA sequencing results. We obtained a number of genes that are both the targets of differentially expressed miRNAs and are also differentially expressed in the pulmonary artery tissues of AS broilers and normal broilers. The general process of association analysis is shown in Additional file 8: Figure S3.

#### Validation of miRNA expression using qRT-PCR

To validate the accuracy of the miRNA deep sequencing by the Illumina high throughput sequencing technology, 4 miRNAs were randomly selected to perform real-time quantitative PCR (qRT-PCR). Total RNA per sample was reverse transcribed to cDNA (miScript Reverse Transcription kit (Qiagen, Valencia, CA, USA)). The primers of each miRNA were designed using primer 5.0 and are shown in Additional file 9: Table S6. The miScript SYBR Green PCR kit (Qiagen, Valencia, CA, USA) was used in each qPCR reaction to determine the expression of each miRNA, whereas the qRT-PCR program was performed using the Applied Biosystems Stepone Plus system (ABI, USA). All reactions were performed in biological triplicates. The corresponding expression level of each miRNA was calculated using the comparative 2−∆Ct method, as well as the fold change.

#### Histopathological examination for the pulmonary artery

To evaluate the histopathological changes in the pulmonary arteries of broilers with AS, pulmonary arteries, which were stored in 10% formaldehyde, were transferred into 4% PFA for more than 24 hours to thoroughly fix the structure of the tissue. Pulmonary artery tissues were placed in an ascending gradient of ethanol (70%-99.5%) for dehydration. They were rendered transparent by dipping in xylene three times (for 4 minutes, 2 minutes, and 30 seconds). Next, they were placed into two beakers filled with paraffin for 1 hour. Finally, the arteries were routinely sectioned and stained with hematoxylin and eosin. The stained sections were examined via microscopy at 200X magnification, and the sections were photographed.

## SUPPLEMENTARY MATERIALS TABLES















## Abbreviations

AS: ascites syndrome
PH: pulmonary hypertension
HIF1α: Hypoxia Inducible Factor 1α
NHE1: Na +/H+ exchanger1
STAT5: Signal Transducer And Activator Of Transcription 5
CD44: cluster differentiation 44
TRAF2: TNF receptor-associated factor 2
CDK2AP1: cyclin-dependent kinase 2-associated protein 1
LZTFL1: leucine zipper transcription factor-like 1
JAZF1: JAZF zinc finger 1
PEBP1: Phosphatidylethanolamine-binding protein 1
LRP1B: low density lipoprotein receptor-related protein 1
RPS14: ribosomal protein S14
THBS2: thrombospondin 2
MEOX2: mesenchyme homeobox 2
IRF8: interferon regulatory factor 8
MAP3K8: mitogen-activated protein kinase kinase kinase 8
IL-1BETA: Interleukin-1 beta
TNFRSF1B: Tumor Necrosis Factor Receptor Superfamily Member
