# Supplementary material for: Dysregulated expression of microRNAs and mRNAs in pulmonary artery remodeling in ascites syndrome in broiler chickens
Source: Oncotarget. 2016 Oct 25;8(2):1993–2007. doi: 10.18632/oncotarget.12888 (PMC5356772; doi:10.18632/oncotarget.12888)
Supplement: Supplementary file 1 [file oncotarget-08-1993-s001.pdf]

Dysregulated expression of microRNAs and mRNAs in pulmonary artery remodeling in ascites syndrome in broiler chickens

Supplementary Material

Additional file 1: Table S1. Known miRNA and novel miRNA analysis.

Additional file 2: Table S2. Identified target genes of differentially expressed known and novel miRNAs in the pulmonary arteries of broiler chickens.

Additional file 3: Table S3. Databases of KEGG pathways for the predicted target genes of miRNAs.

Additional file 4: Table S4. Twenty-nine differentially expressed miRNAs with the corresponding target genes by miRNA-mRNA association analysis.

Additional file 5: Table S5. Eight-hundred-ninety-five differentially expressed genes with corresponding regulatory miRNAs by miRNA-mRNA association analysis.

Additional file 9: Table S6. Primers of four miRNAs for qRT-PCR validation.

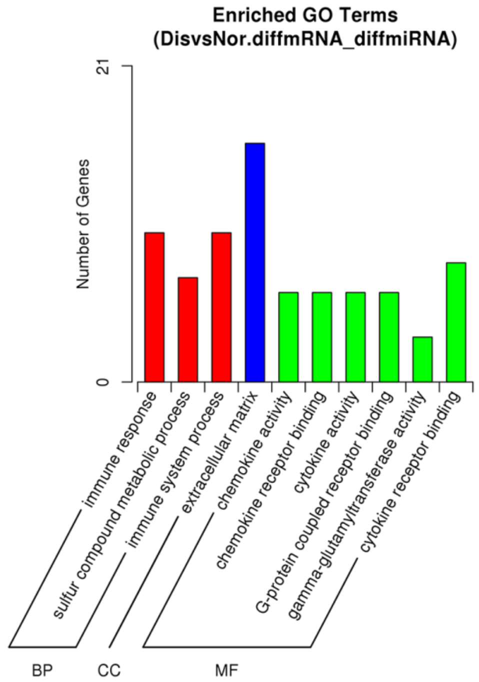

Additional file 6: Figure S1. Enriched GO terms for the genes selected by differentially expressed miRNAs and mRNA association analysis. BP, Biological Process; CC: Cellular Component; MF: Molecular Function; the y-axis indicates targeted gene numbers corresponding to the GO terms.

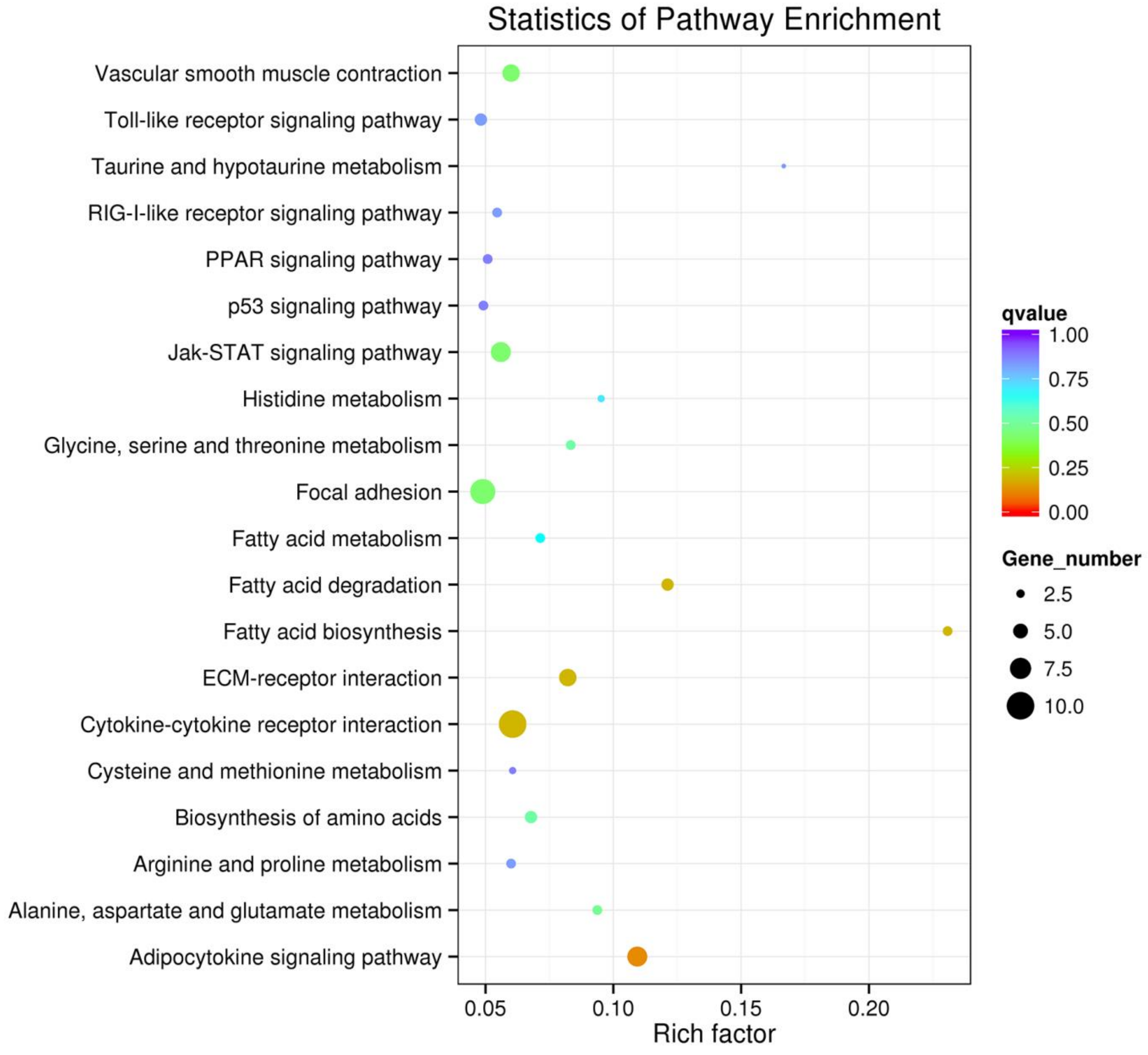

Additional file 7: Figure S2. KEGG analysis with the 20 most enriched pathways for the genes selected using the differentially expressed miRNAs and mRNA association analysis. The coloring of the q-values indicates the significance of the rich factor; the circle indicates the target genes that are involved, and the size is proportional to the gene number.

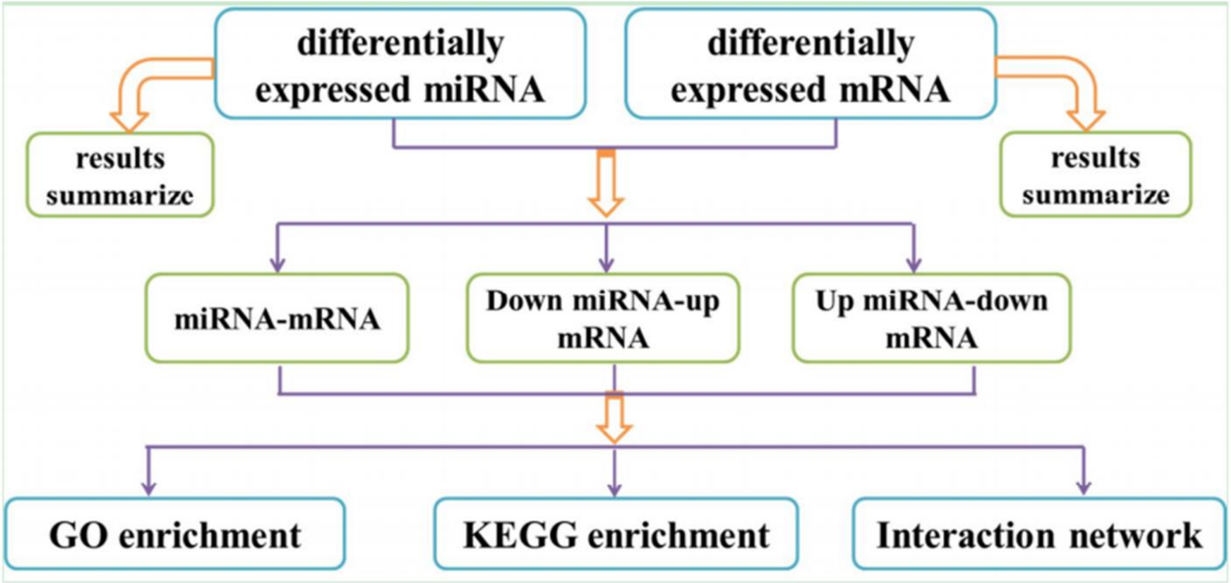

Additional file 8: Figure S3. Biological information analysis process for miRNA-mRNA association analysis.
